# Remote ischemic conditioning combined with transcutaneous vagus nerve stimulation reduces reperfusion injuries and improves cardiac function in acute ST-segment elevation myocardial infarctions

**DOI:** 10.1186/s12872-026-05522-4

**Published:** 2026-01-15

**Authors:** Ling Wang, Zhi-chuan Zhuang, Yin-yin Wu, Jin-tao Yang, Li-li Liu

**Affiliations:** 1 Department of Cardiovascular Medicine, Quanzhou First Hospital Affiliated to Fujian Medical University, Quanzhou, 362000 China; 2 The School of Clinical Medicine, Fujian Medical University, Fuzhou, 362000 China; 3 Department of Endocrinology, Jinjiang Hospital, Jinjiang, Fujian Province 362200 China

**Keywords:** Transcutaneous vagus nerve stimulation, Remote ischemic conditioning, Acute ST-segment elevation myocardial infarction, Ischemic reperfusion injury

## Abstract

**Objective:**

To investigate whether transcutaneous vagus nerve stimulation (TVNS) combined with left upper limb remote ischemic conditioning (RIC) before primary percutaneous coronary intervention (PCI) in patients with an acute ST-segment elevation myocardial infarction (STEMI) can reduce reperfusion injury and improve cardiac function.

**Methods:**

One hundred and thirty-two patients with STEMI were randomly assigned to three different groups—Control, TVNS, and Combined (RIC combined with TVNS) before PCI. The groups were compared by calculating and comparing the area under curve (AUC) of a CK-MB test within 72 h of incidence, the severity of ventricular arrhythmias (VAs) within 24 h after reperfusion, and left ventricular ejection fraction (LVEF) via echocardiography seven days after reperfusion.

**Results:**

Compared with the control group, the combined group demonstrated significantly decreased areas under the curve (AUCs). Specifically, the incidence of VAs within 24 h after reperfusion and BNP levels seven days post-reperfusion were significantly reduced, while the LVEF values at seven days exhibited a significant improvement (*P* < 0.05).In contrast, when compared with the control group, the group receiving only TVNS showed a reduction in the incidence of VAs within 24 h after reperfusion. Although the AUC test presented a decreasing trend, the difference was not statistically significant. Additionally, BNP levels and LVEF values in this group did not show significant improvements (*P* > 0.05).

**Conclusions:**

When administered alone, TVNS treatment significantly reduced the incidence of ventricular arrhythmias (VAs) after reperfusion. However, in the acute phase post-reperfusion, there was no significant difference in myocardial infarct size or left ventricular systolic function, despite a tendency towards improvement. In contrast, the combination of TVNS and RIC treatment led to improvements in left ventricular systolic function, reductions in myocardial infarct size, and decreases in the incidence of VAs.

## Introduction

The most effective treatment for acute myocardial infarction is timely and effective reperfusion therapy [1]. However, reperfusion injury may be one of the most important factors affecting the effectiveness of reperfusion therapy, often manifested as ventricular arrhythmias, impaired myocardial contraction and relaxation function, and coronary microvascular obstruction. It’s been nearly 30 years since the concept of “remote ischemic conditioning (RIC)” was proposed by short repetitive cycles of ischemia/reperfusion on an extremity which attenuates infarct size and improves the prognosis of patients with reperfused myocardial infarction [2, 3]. RIC has been proved to be a a systemic phenomenon not only the heart can be protected also other organs [4]. Cardioprotection by RIC involves neuronal and humoral pathways and, of note, the spleen as a neuro-humoral interface/relay organ [5]. Currently, RIC of the heart has been established in many experimental studies and translated to patients[3]. However, the protective effects of RIC in patients with acute myocardial infarction remain equivocal [6]. In patients with STEMI undergoing PCI, an initial trial (CONDI 1) reported myocardial salvage, but the subsequent trials were equivocal – the larger ERIC-PPCI/CONDI 2 trial was neutral with remote ischemic conditioning at the upper limb, but the smaller RIC-STEMI trial with remote ischemic conditioning at the lower limb demonstrated reduced mortality and hospitalization for heart failure [3].

Since 2005, a substantial number of animal studies have demonstrated that implementing low-level vagus nerve stimulation (LL-VNS), also referred to as cervical vagus nerve stimulation, prior to and during ischemia-reperfusion can effectively reduce the incidence of ventricular arrhythmia and myocardial infarction size, as well as lower inflammation marker levels [7]. Recent studies have revealed that the only area where the vagus nerve is located on the surface of mammalian bodies is the auricular region [8]. TVNS, which stimulates the peripheral vagus nerve in the auricular region, can induce corresponding changes in the central nervous system and produce effects similar to those of LL - VNS [9]. In comparison to LL - VNS, TVNS is less invasive and has been verified for safety in clinical use, rendering it more suitable for clinical treatment [10]. In recent years, numerous animal experiments and some randomized controlled trial(RCT)clinical trials have confirmed that low-level TVNS (LL-TVNS) can reduce ischemia-reperfusion injury in acute STEMI patients, suppress reperfusion ventricular arrhythmia, improve heart failure [10, 11], and inhibit the occurrence of atrial fibrillation [12]. However, these findings needs to be further validated in larger - scale and more comprehensive studies [13].

In summary, currently, there is insufficient clinical evidence to support the exclusive use of TVNS or RIC for the treatment of STEMI patients. The disappointing clinical translation outcomes are partly due to the multifactorial nature of AMI, comorbidities in patients, the timing of interventions, and the redundancy of cell death pathways during IRI. Since these cardioprotective strategies act with inconsistent mechanisms, combining multiple cardioprotective interventions targeting different pathways and cell types, which may lead to additive or synergistic effects, might be essential to achieve robust cardioprotection in clinical settings. This study aims to investigate the effect of TVNS combined with RIC treatment on the ischemia-reperfusion prognosis of acute STEMI patients by conducting non-invasive TVNS treatment combined with left upper limb ischemia treatment before reperfusion.

## Materials and methods

### Research subjects and inclusion criteria

The first trial registration date was August 22, 2021. The registration number for the clinical trial is ChiCTR2100050204. Patients admitted with STEMI to the cardiology department of the First Hospital of Quanzhou from February 2022 to October 2022 were continuously enrolled, if they met the following inclusion criteria: (1) The diagnosis of acute STEMI according to the “2019 Diagnosis and Treatment Guidelines for Acute ST-Segment Elevation Myocardial Infarction” [14]. (2) PCI treatment was performed at the First Hospital of Quanzhou. (3) Preoperative and postoperative treatments were meticulously carried out in strict accordance with the aforementioned guidelines. (4) Onset time was less than 12 h. (5) Age between 18 and 80 years. (6) Patients have informed consent.

Patients were excluded based on the following criteria: (1) History of previous myocardial infarction. (2) Severe heart failure upon admission (left ventricular ejection fraction < 30%). (3) Cardiogenic shock. (4) Ventricular fibrillation. (5) Cardiac arrest. (6) History of severe liver dysfunction. (7) History of chronic kidney failure (glomerular filtration rate < 30 mL/min). (8) History of blood diseases. (9) Left main coronary artery disease. (10) Multivessel disease.

### Research methods

All patients were administered atorvastatin (40 mg) and either aspirin enteric-coated tablets (300 mg), ticagrelor (180 mg), or clopidogrel (300 mg) immediately after arrival or admission. From the second day, patients were given aspirin enteric-coated tablets (100 mg)/ticagrelor (90 mg) twice daily or clopidogrel 75 mg once daily. Beta-blockers, ACEIs or ARBs, statins, diuretics, and other medications were prescribed as needed. All other drug treatments were strictly implemented in accordance with the guidelines.

Before entering the catheterization room, all patients were randomly allocated to one of three groups in blocks of 4 according to a computer - generated randomization list: Control, TVNS, and Combined (RIC combined with TVNS).All three groups underwent routine coronary angiography, direct PCI, and received standardized treatment with coronary heart disease medications as per the guidelines. In the TVNS group, an ear vagus nerve stimulator (SDZIIb, Huatuo brand) was utilized to deliver low - frequency square wave pulses (20 Hz, pulse width 1 ms) at the tragus of the left ear. The current intensity was gradually increased until sinus bradycardia occurred. The minimum current required to slow the sinus rate was defined as the stimulation threshold, and the selected intensity was half of the stimulation threshold. Refer to Yu L’ protocol [11], the stimulation was started with a duty cycle of 5 s on and 5 s off and last for 120 min upon the patient ‘s arrival at the catheterization room. For the RIC process, a mercury sphygmomanometer cuff was placed on the left upper arm. This involved four alternating cycles of cuff inflation to 200 mmHg for 5 minutes followed by deflation for 5 minutes .In the RIC combined with TVNS group, both procedures were simultaneously performed on the patients. The control group received only routine PCI without any additional interventions.

This study was approved by the Ethics Committee of the First Hospital of Quanzhou, and all patients provided informed consent.

### Laboratory tests and examinations

The CK-MB level was measured from venous blood collected at admission and at 2, 12, 24, 48, and 72 h after reperfusion, using a Beckman Coulter AU5800 automatic biochemical analyzer. BNP level was measured from venous blood collected at admission, 24 h after reperfusion, and 7 days after reperfusion using the AxSYM automatic immunoassay analyzer (Abbott Laboratories, U.S.A). The detection principle was the particle capture immunoluminometric assay with a quantitative detection range of 0-4000 pg/mL. The normal value is < 100 pg/mL for adults.

Color Doppler ultrasound examination: All enrolled patients underwent cardiac color Doppler ultrasound examination 7 days after reperfusion to evaluate left ventricular ejection fraction (LVEF) as an assessment of left ventricular systolic function, using a Phillips IE10 color Doppler ultrasound diagnostic instrument. LVEF was measured using Simpson’s method.

Dynamic electrocardiogram: A 12-lead remote dynamic electrocardiogram was worn by patients starting from reperfusion and was continued for 24 h. A LePu dynamic electrocardiogram recorder was used, and the system automatically analyzed the severity of ventricular arrhythmias (VAs), the number of ventricular tachycardia (VT) episodes, and the total number of ventricular premature beats (VPB).

### Statistical analysis

Statistical analysis was performed using Graphpad Prism to calculate the area under the curve (AUC) of the CK-MB levels at various time points within 72 h post - treatment. For other data, SPSS 25.0 was employed. Continuous data underwent normality tests, and Variables with a normal distribution were presented as mean ± standard deviation (SD). ANOVA was utilized to compare age, CK-MB AUC, and LVEF among the three groups, followed by Bonferroni correction for pairwise comparisons. Non-normally distributed variables were presented as median (interquartile range) : M (P25, P75) and analyzed via the Kruskal-Wallis test. Pairwise comparisons were also adjusted with the Bonferroni correction. This approach was applied to baseline data such as the severity of VAs, ischemic time, and distribution of culprit vessels. Quantitative data were presented as percentages and analyzed using the chi-square test for differences in the distribution of gender, smoking history, hypertension, diabetes, and other variables. In cases where the assumptions for the chi - square test were not met, Fisher’s exact test was employed. All statistical tests were two-sided, and *P* < 0.05 was considered statistically significant.

## Results

### Comparison of general data of enrolled patients

A total of 132 STEMI patients were initially enrolled in this study. These patients were randomly allocated into three groups: the control group (*n* = 44), the TVNS group (*n* = 44), and the combined group (*n* = 44). During coronary angiography, five patients were identified as having left main trunk lesions or multivessel disease. Moreover, four patients either succumbed to their conditions after admission or were discharged at their own initiative within five days of admission.These nine patients were excluded from the study. Ultimately, the remaining patients in the control group (*n* = 42), the TVNS group (*n* = 40), and the combined group (*n* = 41) proceeded to participate in the experiment. There was no statistically significant difference among the three groups in terms of age, gender, history of hypertension, diabetes, dyslipidemia, smoking history, total ischemia time, distribution of ischemia time, culprit vessel, post-PCI TIMI flow grade or prescription medications (Table 1).

**Table 1:** General information of the control group, TVNS group, and combination group [ X̅±S, n(%), M(P25,P75)]

General information	Control(*n* = 42)	TVNS(*n* = 40)	*Combination*(*n* = 41)	*P*
Age	59 ± 9	58 ± 10	57 ± 8	0.89
Male	37(88.1)	36(90.0)	37(90.2)	1.00
Smoking	30(71.4)	24(60.0)	23(56.1)	0.33
Hypertension	22(52.4)	12(30.0)	16(39.0)	0.12
Diabetes	6(14.3)	4(10.0)	7(17.1)	0.68
Dyslipidemia	27(64.3)	29(72.5)	27(65.9)	0.72
Ischemic time	4.3(2.0, 7.0)	4.0(3.0, 7.0)	4.0(3.0, 7.0)	0.83
Distribution of ischemic time				
T ≤ 4 h	23(54.8)	22(55.0)	21(51.2)	
4 < T ≤ 8	10(23.8)	12(30.0)	16(39.0)	0.49
8 < T ≤ 12	9(21.4)	6(15.0)	4(9.8)	
culprit vessel				
LAD	26(61.9)	18(45.0)	20(48.8)	
RCA	14(33.3)	20(50.0)	16(39.0)	0.36
LCX	2(4.8)	2(5.0)	5(12.2)	
TIMI flow grade 3 post-PCI	39(92.9)	36(90.0)	37(90.2)	0.86
Oral medicines after PCI				
Aspirin	42(100)	40(100)	41(100)	1.00
Clopidogrel	15(35.7)	11(27.5)	14(34.1)	0.72
ticagrelor	27(64.3)	29(72.5)	27(65.9)	0.72
Beta-blockers	38(91.6)	36(91.1)	37(89.6)	0.83
ACE inhibitors or ARBs	34(81.6)	33(83.3)	34(83.3)	0.90

*T*  the duration of myocardial ischemia, *LAD*  Left Anterior Descending artery, *RCA*  Right Coronary Artery, *LCX*  Left Circumflex Artery, *PCI*  percutaneous coronary intervention

TIMI blood flow grade 3 = Forward blood flow (contrast agent) rapidly and completely fills the distal blood vessels

### Comparison of CK-MB AUC within 72 h after reperfusion among the three groups

**Fig. 1:**
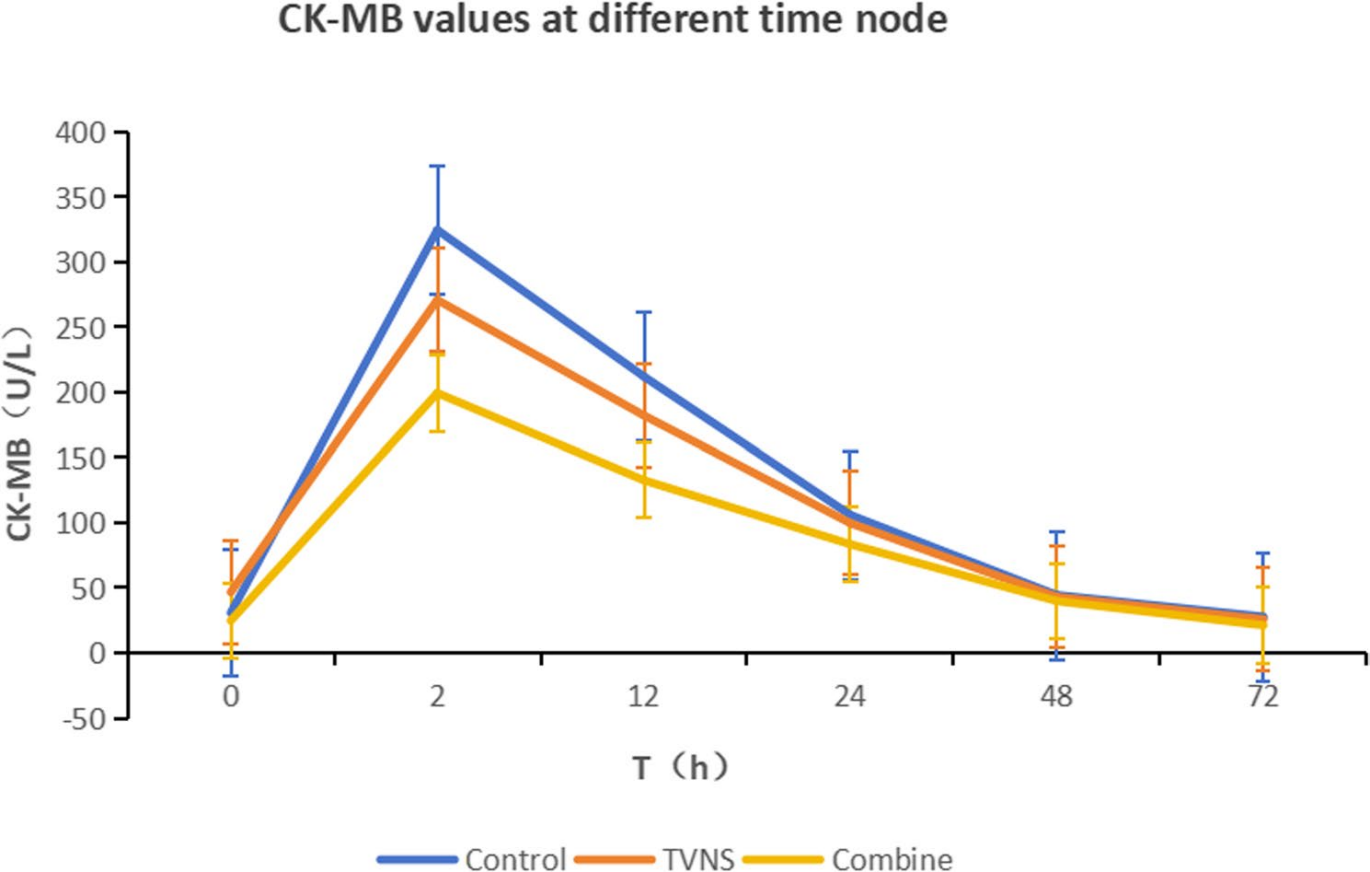
Comparison of creatine kinase-MB (CK-MB) (U/L) levels at different time points after reperfusion among the three group (X̅±S)

**Fig. 2:**
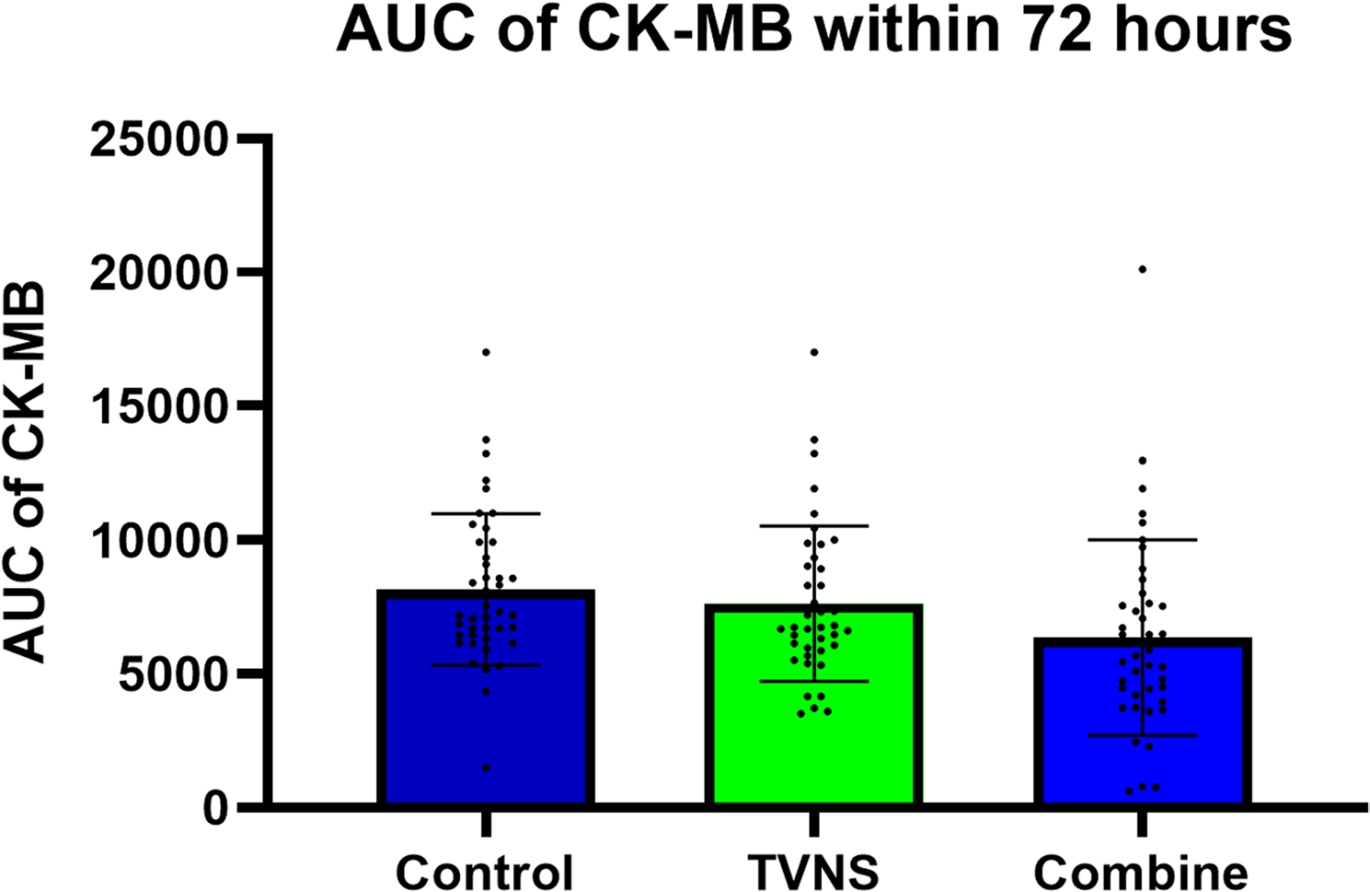
Comparison of the AUC values of CK-MB within 72 hours after reperfusion in the three groups (X̅±S)

The analysis of CK-MB AUC within 72 h after reperfusion demonstrated a statistically significant difference among the control group, the TVNS group, and the combination group (8154.8 ± 436.2 vs. 7627.6 ± 459.4 vs. 6356.0 ± 571.7, F = 3.553, *P* < 0.05). Post-hoc pairwise comparisons revealed that the combination group had a significantly lower CK - MB AUC within 72 h compared with the control group. When the combination group was compared with the TVNS group, the CK - MB AUC within 72 h was slightly decreased, yet the difference did not reach statistical significance. The TVNS group also showed a slight reduction in the CK-MB AUC within 72 h compared with the control group, but the difference was not statistically significant (Figs. 1 and 2).

### Comparison of ventricular arrhythmias (VAs) within 24 h after reperfusion

**Fig. 3:**
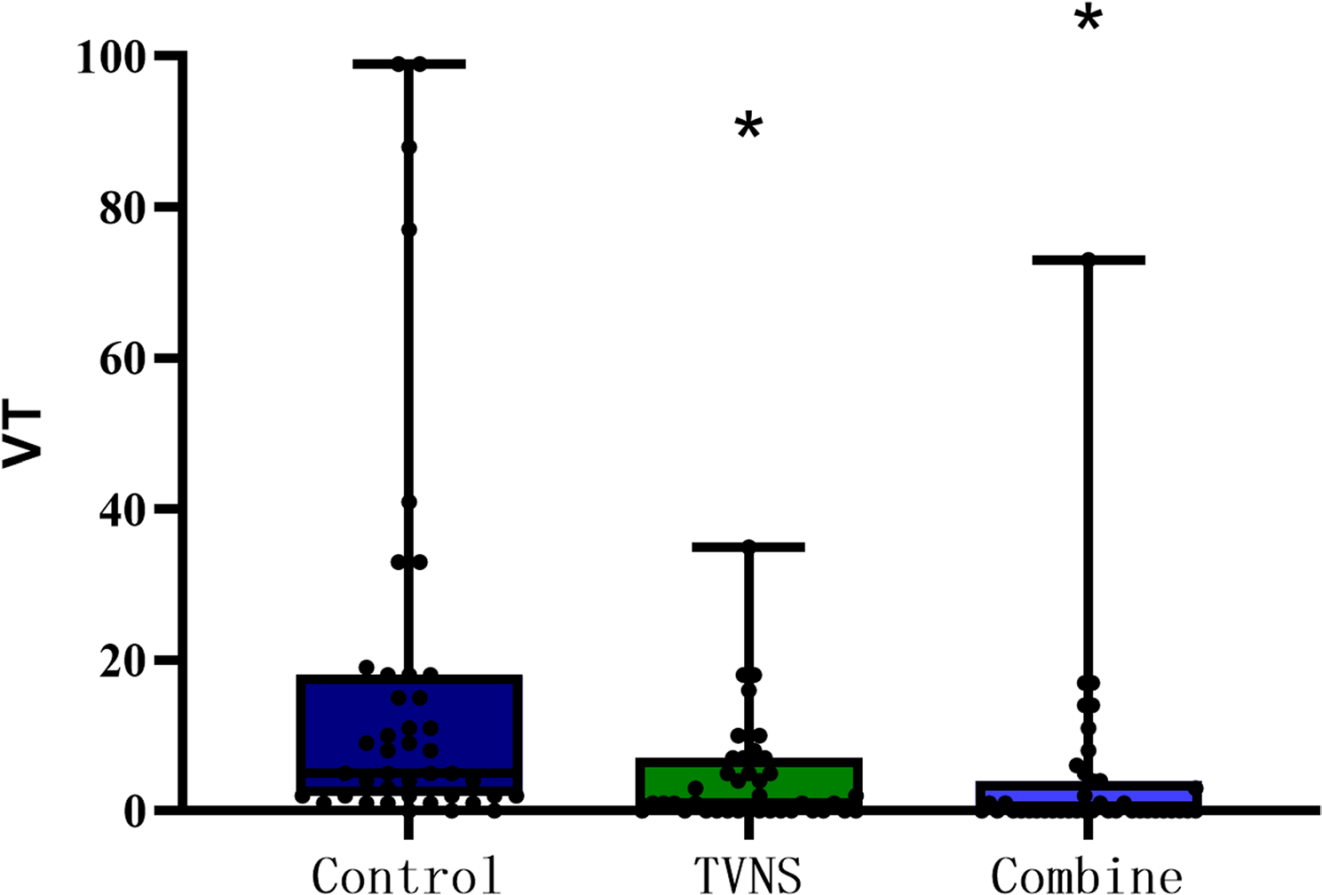
Number of ventricular tachycardia (VT) within 24 hours after reperfusion among the three groups M(P25,P75)

**Fig. 4:**
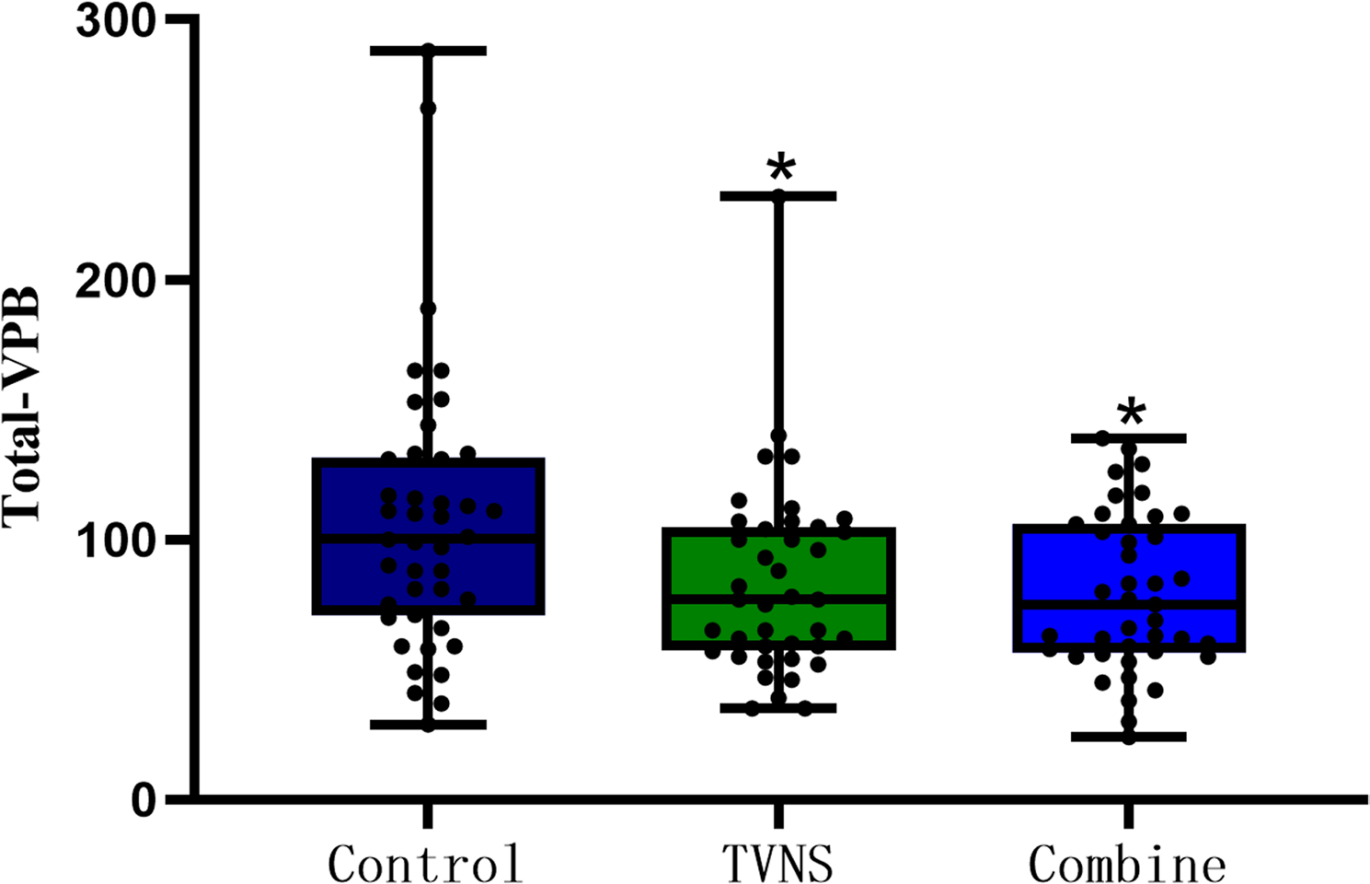
Number of ventricular premature beats (VPBs) in the three groups within 24 hours after reperfusion M(P25, P75)

Analysis of remote dynamic electrocardiograms within 24 h after reperfusion revealed statistically significant differences in the total number of VTs and VPBs among the control group, the TVNS group, and the combination group (*P* < 0.05). Post-hoc pairwise comparisons demonstrated that, within 24 h after reperfusion, both the TVNS group and the combination group had a significantly smaller number of VPBs and VTs compared with the control group. However, no statistically significant difference was observed between the combination group and the TVNS group. (Table 2; Figs. 3 and 4).

**Table 2:** Statistical analysis of the incidence of ventricular arrhythmias within 24 hours after reperfusion among the three groups [M (P25, P75)]

	VT number	Total VPB number
Control(42)	5(2,18)	111(71,145)
TVNS(40)	1(0,7)*	77(58,105)*
Combination(41)	0(0,4)*	75(57,106)*
H value	23.394	11.416
*P* value	< 0.001	0.003

*VT* ventricular tachycardia, *VPB* ventricular premature beat

* indicates a statistical significance of *P* < 0.05 compared to the control group

### Comparison of BNP levels among the three groups at admission, 24 h, and Seven days after reperfusion

**Fig. 5:**
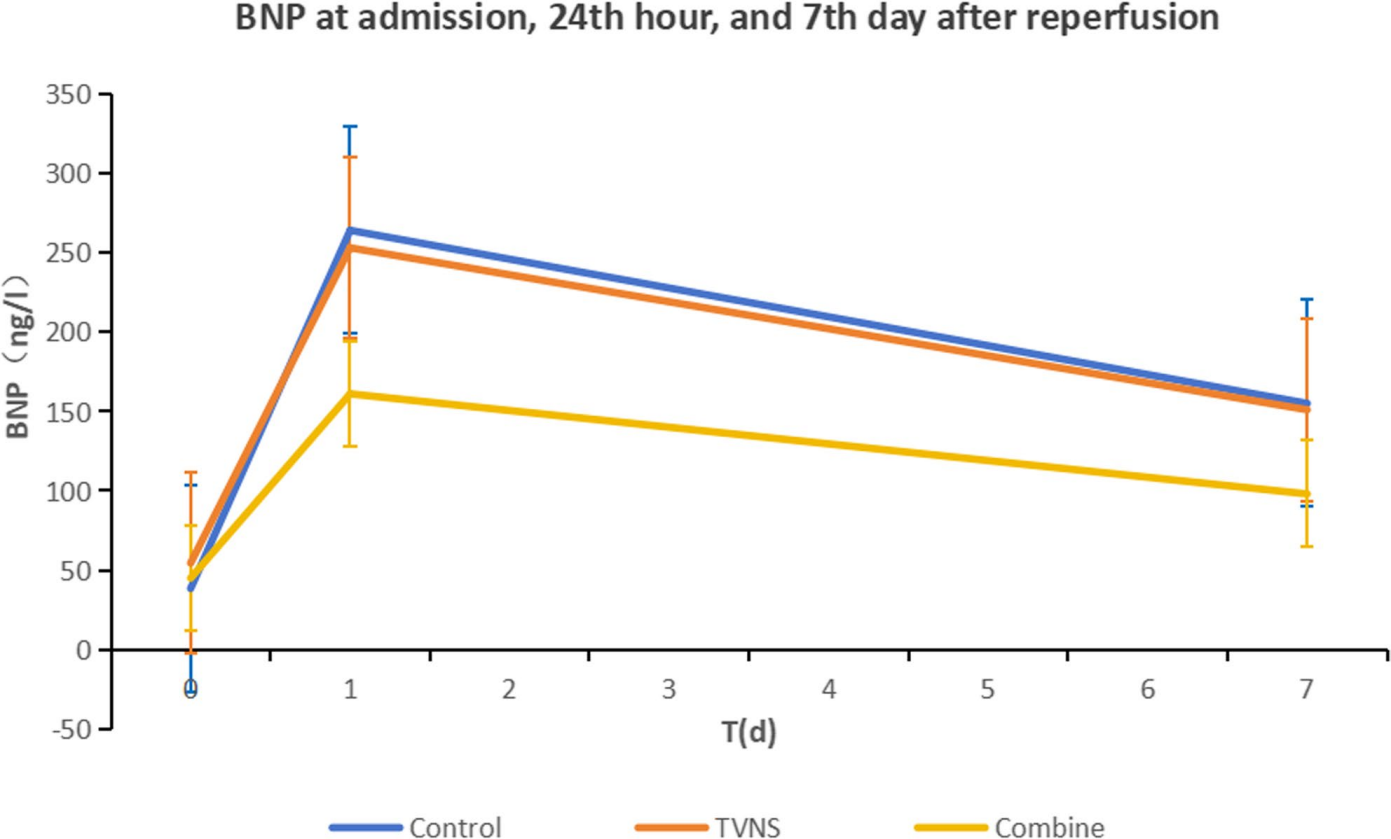
Trends in BNP levels among the three groups at admission, 24 hours after reperfusion, and 7 days after reperfusion M(P25, P75)

No differences were detected in BNP levels among the control group, TVNS group, and combination group at admission (*P* > 0.05). However, statistically significant differences in BNP levels were observed 24 h and 7 days after reperfusion among the three groups (*P* < 0.05). Subsequent post - hoc pairwise comparisons demonstrated that, compared with the control group, the combination group had significantly decreased BNP levels 24 h and 7 days after reperfusion. Moreover, when compared with the TVNS group, the combination group also showed a statistically significant reduction in BNP levels 7 days after reperfusion. However, no statistically significant differences in BNP levels were found between the TVNS group and the control group 24 h and 7 days after reperfusion .(Table 3; Fig. 5).

**Table 3:** Comparison and analysis of BNP levels among the three groups at admission, 24 hours after reperfusion, and 7 days after reperfusion. [M(P25,P75)]

	At admission BNP (pg/ml)	24 h after reperfusion BNP (pg/ml)	7 days after reperfusion BNP (pg/ml)
Control	38.5(15.8,104.5)	264.0(160.0,471.8)	155.0(83.0,243.8)
TVNS	54.5(23.0,107.0)	253.0(161.8,370.3) ^&^	151.0(98.0,232.8) ^&^
Combination	45.0(25.5,109.5)	161.0(79.5,270.0)*^#^	98.0(44.5,158.0)*^#^
H	0.924	8.921	10.992
*P*	0.63	0.012	0.004

*BNP* B-type natriuretic peptide

^*^P < 0.05 compared to the control group

^#^*P* < 0.05 compared to the TVNS group

^&^*P* >0.05 compared to the control group

### Comparison of left ventricular ejection fraction (LVEF) values among the three groups after 7 days of reperfusion

Seven days after reperfusion, a comparison of LVEF values was carried out. The results demonstrated that, in contrast to the control group, the combination group exhibited a statistically significant improvement in LVEF values. When compared with the TVNS group, the combination group showed a slight improvement in LVEF values seven days after reperfusion, yet the difference did not reach statistical significance. Similarly, the TVNS group showed a marginal improvement in LVEF values compared with the control group seven days after reperfusion, but this difference was also not statistically significant. (Table 4).

**Table 4:** Comparison of LVEF among the three groups at 7 days after reperfusion(X̅ ± S)

	LVEF(%)7 days after reperfusion
Control	53.6 ± 1.8 (*n* = 42)
TVNS	55.2 ± 1.5 ^#^ (*n* = 40)
Combination	59.4 ± 1.1* (*n* = 41)
F	44.117
*P*	0.02

*LVEF* left ventricular ejection fraction

* indicates a statistically significant difference compared to the control group (*P* < 0.05)

^#^ indicates no statistically significant difference compared to the control group (*P* > 0.05)

### Safety analysis

During and after the stimulation procedure, neither the transcutaneous vagus nerve stimulation (TVNS) group nor the combination group experienced any adverse reactions, including dizziness, palpitations, or tinnitus. In the combination group during the remote ischemic conditioning (RIC) treatment, patients reported numbness in the left upper limb during the operation. However, this symptom could be alleviated upon completion of the treatment, and there was no significant discomfort such as swelling or pain in the left upper limb. All patients in the TVNS group and the combination group were followed up for one month after the surgery. The results showed that no adverse reactions related to the experimental procedures occurred.

## Discussion

This study innovatively integrated the two treatments to explore their clinical significance in mitigating reperfusion injury in acute STEMI. The key findings of this study are as follows: (1) When applied alone, TVNS treatment can significantly reduce the incidence of post-reperfusion VAs. However, in the acute stage after reperfusion, there is no significant difference in myocardial infarction size and left ventricular systolic function.(2) The synergistic application of TVNS and RIC was observed to enhance cardiac systolic performance, reduce the size of myocardial infarction, and decrease the incidence of VAs.

RIC was once regarded as the most promising cardioprotective strategy for improving clinical outcomes following STEMI [3]. Despite the initial promise demonstrated by numerous preclinical and smaller proof-of -principle clinical trials, the latest findings from the CONDI-2/ERIC-PPCI trial provide definitive and conclusive evidence that RIC offers no benefits in terms of either MI size or clinical outcomes in STEMI patients treated by PCI. A multitarget cardioprotective therapy might be essential, which combines different types of cardioprotective strategies [6]. The combination of TVNS and RIC has the potential to synergize their protective effects against I/R injury.

LL-VNS is a relatively novel interventional measure for cardioprotection. Current research has confirmed that LL-VNS, even in the absence of heart rate reduction, can protect the myocardium through multiple mechanisms. These include reducing inflammation mediators, regulating myocardial cell apoptosis, and suppressing sympathetic nervous activity [15–18]. Sympathetic activation has been demonstrated to increase the susceptibility of the heart to ischemia or reperfusion-related VAs [5]. High levels of vagal activity exert powerful anti-arrhythmic effects [19]. Recently, Yu et al. found that low-level transcutaneous vagus nerve stimulation (LL - TVNS) reduces cardiac autonomic remodeling and the incidence of animal experiments and clinical randomized studies, respectively [11, 20].

This study is partially consistent with previous findings: LL-TVNS can significantly attenuate the occurrence of VT and VPB within 24 h after reperfusion [11]. Regarding reperfusion ventricular arrhythmias, the combination of RIC and TVNS shows a similar effect to that of TVNS alone. Specifically, it did not produce a significant additive effect in reducing the occurrence of VT and VPB after reperfusion.

The level of cardiac enzymes can serve as an indicator to measure the degree of myocardial damage and indirectly evaluate the size of myocardial infarction. Yu L et al. discovered that in patients with STEMI, LL-TVNS administered prior to surgery could reduce AUC of CK-MB within 72 h after surgery, and improve BNP levels and LVEF post-surgery [11].

However, the results of this study did not fully reproduce those of the clinical trial by Yu L et al. Although TVNS treatment exhibited a tendency to decrease the CK-MB AUC after reperfusion and enhance cardiac function, the effect was not statistically significant. It remains unclear whether this was due to the small sample size or other biases that precluded the achievement of clear-cut clinical benefits. Nevertheless, based on our research, the post - hoc power was calculated to be 98% with a two - sided α error of 5%. Notably, the results of this study revealed, compared with the control group, the combination group had significantly reduced AUCs of CK-MB within 72 h after myocardial reperfusion, improved BNP levels and LVEF 24 h and 7 days after reperfusion.

It is postulated that the combination of transcutaneous vagus nerve stimulation (TVNS) and remote ischemic conditioning (RIC) treatment exerts a distinct synergistic effect in alleviating myocardial reperfusion injury and reducing infarct size in patients with ST - segment elevation myocardial infarction (STEMI). Although the clinical translation of RIC has been underwhelming, in animal models, the mechanisms of RIC involve a complex interaction among neural, humoral, immune, and mitochondrial pathways, ultimately resulting in systemic protection against ischemia - reperfusion injury [4, 5]. Key mediators in this process include adenosine, nitric oxide, anti - inflammatory cytokines, and exosomes. Moreover, the spleen and the vagus nerve play pivotal roles in signal transduction [6]. Preclinical studies have validated that the cardioprotection induced by RIC can modulate the intrinsic cardiac nerves and the peripheral sensory afferent nerves in the limb and the vagus nerve, respectively. Additionally, it has been demonstrated that RIC is mediated via the vagus - spleen axis [3]. Meanwhile, TVNS is also hypothesized to be mediated by the vagus nerve afferent fibers [11]. Since the two interventions are founded on similar biological mechanisms, this appears to furnish a mechanistic foundation for their synergistic effects.

Based on the current findings, the collaborative mechanism of the combination therapy can be manifested in the following aspects:1. Autonomic Nervous System Modulation: TVNS effectively enhances the vagal tone while concurrently suppressing excessive sympathetic activity [21, 22]. RIC, on the other hand, attenuates the activation of the sympathetic nervous system, thereby playing a crucial role in stabilizing the autonomic balance [3, 23]. 2. Anti - Inflammatory Effects: TVNS activates the cholinergic anti-inflammatory pathway. Specifically, it reduces the levels of pro-inflammatory cytokines, such as tumor necrosis factor - α (TNF - α) and interleukin − 6 (IL − 6), through the activation of α − 7 nicotinic acetylcholine receptors [21]. In contrast, RIC suppresses systemic inflammation by precisely modulating immune cell activity and cytokine release [4, 23]0.3. Antioxidant Effects: TVNS mitigates oxidative stress by augmenting the activity of antioxidant enzymes, for example, superoxide dismutase, and concomitantly decreasing the production of reactive oxygen species (ROS) [24, 25]. Similarly, RIC activates endogenous antioxidant pathways, notably the nuclear factor erythroid 2-related factor 2/antioxidant response element (Nrf2/ARE) signaling pathway [4, 23]0.4. Anti - Apoptotic and Pro-Survival Signaling: TVNS activates pro-survival pathways, such as the phosphatidylinositol 3-kinase/protein kinase B (PI3K/Akt) and extracellular signal-regulated kinase (ERK) pathways, and simultaneously inhibits apoptosis [24]. RIC, in parallel, triggers the Reperfusion Injury Salvage Kinase (RISK) and Survivor Activating Factor Enhancement (SAFE) pathways, consequently reducing cell death [23].In our perspective, by simultaneously targeting multiple pathways, these therapies can offer more comprehensive and efficacious cardioprotection in the setting of IRI.

In our initial remote ischemic conditioning (RIC) protocol, we selected the upper arm for its convenience. In previous experimental studies, it was demonstrated that the reduction in infarct size achieved by RIC was more pronounced when a larger mass of peripheral tissue underwent ischemia and reperfusion. The stimulus applied to the arm in our trial might have been less effective in providing sufficient cardioprotection due to the lower ischemic tissue burden [26].Although the RIC - STEMI, the only prospective, randomized trial on RIC demonstrating cardioprotection in patients with acute myocardial infarction, performed RIC on the leg, it did not show a reduction in myocardial infarct size [27]. Nevertheless, there remains substantial research evidence to support the use of the arm for RIC [28–30].

Experimental investigations have demonstrated that specific comorbidities, including advanced age and diabetes, along with concomitant medications, can weaken the cardioprotective effects of ischemic conditioning strategies [31, 32]. Aspirin, for instance, interferes with the transfer of cardioprotective factors mediated by platelets. Conversely, ticagrelor promotes the generation of cardioprotective factors that are transported by platelets and plasma. As a result, in clinical trials, the application of dual - platelet inhibition using aspirin and ticagrelor might confound the assessment of the cardioprotective intervention under study [33].Notably, in our study, factors such as age, the presence of diabetes, or the administration of the P2Y12 receptor antagonist, ticagrelor, did not exert any interference with the clinical outcomes among the three groups.

In summary, the results of our trial offer conclusive evidence that TVNS therapy alone can reduce ventricular arrhythmias following reperfusion. Moreover, the combination of TVNS and RIC treatment exerts a synergistic effect in diminishing myocardial reperfusion injury and infarct size in patients with STEMI. The combination therapies involving “TVNS” and “RIC” signify a promising strategy for enhancing cardioprotection.

### Limitations of the study

This study does have several limitations. Firstly, the parameters utilized for TVNS, including frequency, intensity, duration, and the side of stimulation (left ear, right ear, or bilateral ears), are founded on previous research. However, the optimal parameters for LL-TVNS remain undetermined. Patients undergoing TVNS stimulation may experience mild pain, which could potentially trigger a protective response and thus interfere with the test results.Secondly, the parameters chosen for RIC are also based on previous clinical studies, yet there is no conclusive data to firmly support their application. Thirdly, in this study, the myocardial infarction size was solely evaluated by the CK-MB curve within 72 h after PCI. Cardiac magnetic resonance imaging was not performed to more precisely assess the infarction size. Relying on this single method may lead to a lack of accuracy.Fourthly, high-risk patients, such as those with left main or multivessel disease, were excluded from the study. Consequently, it remains unclear whether this intervention is effective for these higher-risk patient populations. Additionally, this study is a single- center investigation with a small sample size and a short follow-up time. As a result, the conclusions drawn are inevitably limited. Further studies with larger sample sizes and multi-center collaborations are essential to validate and refine the conclusions reached.

## Data Availability

The data used to support the findings of this study are available from the corresponding author upon request.

## Abbreviations

AUC: Area under the curve
BNP: B-type natriuretic peptide
CK-MB: Creatine kinase isoenzyme MB
HMGB1: High-mobility group-box 1 protein
IL-1β: Interleukin-1β
IL-6: Interleukin-6
IL-8: Interleukin-8
LAD: Left anterior descending
LCX: Left circumflex
LL-VNS: Low-level vagus nerve stimulation
LVEF: Left ventricular ejection fraction
NO: Nitric oxide
NT-proBNP: N-terminal pro-brain natriuretic peptide
PCI: Percutaneous coronary intervention
RCA: Right coronary artery
RIC: Remote ischemic conditioning
SDF-1α: Stromal cell-derived factor-1α
STEMI: ST-segment elevation myocardial infarction
TNF-α: Tumor necrosis factor–α
TnT: Troponin T
TVNS: Transcutaneous vagus nerve stimulation
VAs: Ventricular arrhythmias
VF: Ventricular fibrillation
VPB: Ventricular premature beat
VT: Ventricular tachycardia
